# Changes in Drug Use Patterns during the COVID-19 Pandemic in Italy: Monitoring a Vulnerable Group by Hair Analysis

**DOI:** 10.3390/ijerph18041967

**Published:** 2021-02-18

**Authors:** Alessio Gili, Mauro Bacci, Kyriaki Aroni, Alessia Nicoletti, Angela Gambelunghe, Isabella Mercurio, Cristiana Gambelunghe

**Affiliations:** 1 Hygiene and Public Health Section, Department of Medicine and Surgery, University of Perugia, Piazza Lucio Severi, 06132 Perugia, Italy; alessio.gili@gmail.com; 2 Forensic Medicine, Forensic Science and Sports Medicine Section, Department of Medicine and Surgery, University of Perugia, Piazza Lucio Severi, 06132 Perugia, Italy; mauro.bacci@unipg.it (M.B.); aroniky@libero.it (K.A.); nicolettialessia8@gmail.com (A.N.); isabmerc@gmail.com (I.M.); 3 Occupational Medicine, Respiratory Diseases and Toxicology Section, Department of Medicine and Surgery, University of Perugia, Piazza Lucio Severi, 06132 Perugia, Italy; angela.gambelunghe@unipg.it

**Keywords:** COVID-19 pandemic, substance abuse disorder, addiction, lockdown, hair analysis

## Abstract

From 22 March until 18 May 2020, a complete lockdown in Italy was ordered as a countermeasure against the COVID-19 pandemic. Social isolation measures affect some populations more than others, and people with drug and/or alcohol disorders (SUDs) are more likely to be adversely affected. This study presents, for the first time, laboratory data on the use of alcohol and drugs in a high-risk population during Italy’s first wave of the COVID-19 pandemic. Thirty subjects with SUDs were monitored for the use of illicit drugs and alcohol every 3 months before, during and after the lockdown, by hair analysis. The number of samples positive for heroin, cocaine, MDMA and cannabis fell considerably during the lockdown and then resumed to pre-lockdown levels when the period of confinement was over. Interestingly, the consumption of benzodiazepines and alcohol followed the opposite trend; both the number of benzodiazepine-positive samples and the level of alcohol consumption increased and remained high, even at the end of the lockdown. The confinement measures produced significant changes in drug/alcohol use patterns, with a shift toward the use of substances that were more easily accessible, used as self-medication for negative feelings, and used to alleviate the effects of abstinence from drugs that were no longer readily available.

## 1. Introduction

The coronavirus disease of 2019 (COVID-19), caused by severe acute respiratory syndrome coronavirus 2 (SARS-CoV-2), was first identified in December 2019 in Wuhan, China, and then declared as a public health emergency of international concern in January 2020, and as a pandemic in March 2020 [1]. The Italian government has implemented various initiatives since 10 March 2020 to prevent or delay the spread of COVID-19, such as the lockdown of social and cultural activities and the partial closure of economic and industrial activities [2,3]. A second government decree, on 22 March 2020, imposed even stricter rules, such as the closure of all non-strategic economic activities, including schools, universities and shops selling non-essential goods [3,4]. People were allowed to leave their homes for specific and documented reasons only [3]. Although these measures were necessary to reduce the pressure on the Italian health system, there are reasons to be concerned, because prolonged home confinement during an epidemic can reduce levels of physical activity and exposure to daylight, and increase the level of stress due to social isolation [5,6]. There are precedents for such measures. Citywide quarantines were also imposed in areas of China and Canada during the 2003 outbreak of SARS, whereas entire villages in many West African countries were quarantined during the 2014 Ebola outbreak [7]. The mental health impact can be broad, extensive and long-lasting [7]. Among the consequences of quarantine are acute stress disorders, anxiety, irritability, poor concentration and indecisiveness, poor work performance, post-traumatic stress disorders, high psychological distress, depressive symptoms and insomnia [8]. In addition, the economic consequences of the COVID-19 outbreak can be particularly dramatic for people in precarious employment or financial conditions, causing them unparalleled distress due to the sheer uncertainty about their futures [9]. Distressed people may seek refuge in inexpensive and readily available addictive substances to allay their negative feelings [10]. This can potentially trigger the development of drug and/or alcohol disorders (SUDs) in high-risk groups as well as a spike in the incidence of SUDs among the general population [11,12,13]. Persons who are isolated and stressed, as much of the population is during a pandemic, frequently turn to such substances to alleviate their negative feelings [14].

This warrants the question of whether the significant increases in alcohol sales (including those by mail) that were observed in many countries compared with those in the same period of the previous year, are due to the pandemic [15]. As an example, a March 2020 study conducted by the Nielsen Company in the USA found a 240% increase in internet alcohol sales, including an increase in the sale of strong liquor (spirits) by 75%, wine by 66% and beer by 42% [16].

Although the literature concerning substance use in the context of COVID-19 is still nascent, past research from other large-scale disasters suggests that, in general, increases in substance use are observed following exposure to a disaster [17].

To the best of our knowledge, there are currently no studies that have documented changes in drug/alcohol consumption during the COVID-19 pandemic with experimental data from a toxicology laboratory. Our study investigated drug and alcohol misuse in 30 subjects with a history of substance and/or alcohol abuse who were monitored every 3 months during the first wave of the COVID-19 pandemic in Italy (before the lockdown, during the lockdown and 3 months after the end of the lockdown) by means of hair analysis, a method that has become widespread in recent years and that provides valuable retrospective information [18]. Depending on hair length, the analysis provides long-term information on drug use, complements other biological matrix analyses and may offer crucial data for evaluating and interpreting the results and reaching conclusions [18]. Collection is non-invasive, and storage is easy at room temperature [18].

We believe that the results from this study can contribute to the discussion on the risk of substance/alcohol abuse during the COVID-19 pandemic.

## 2. Materials and Methods

### 2.1. Patients and Sample Collection

Laboratory procedures were conducted in accordance with the Helsinki Declaration of 1975 (revised 1983) and approved by the Bioethics Review Board of the University of Perugia (Protocol 2012-006R). All participants provided informed consent. The study material consisted of 3 cm hair samples collected from 30 patients (aged 18–48 y; 17 males; 13 females) from urban areas of central Italy. Patients were voluntarily registered with the Public Service for Drug Dependence Treatment, where they received counselling therapy without the use of opiate substitution drugs, such as methadone or buprenorphine. Drug and/or alcohol disorders were diagnosed according to the Diagnostic and Statistical Manual of Mental Disorders (DSM-5) criteria. The subjects were regularly monitored for drug/alcohol consumption at the toxicology laboratory via hair analysis every three months. All patients submitted lists of prescription medications for any other pathologies. Benzodiazepines (BZDs) were not present in the therapeutic plan as medications.

### 2.2. Preparation and Analysis of Hair Samples

All hair samples were prepared, extracted and derivatized by the fully validated, previously described methods [18].

Opiates (codeine, 6-acetylmorphine, morphine), cocaine (and its metabolite, benzoylecgonine), cannabinoids (D9-tetrahydrocannabinol and its metabolite, 11-nor-9-carboxy-D9-tetrahydrocannabinol), amphetamine, methamphetamine, MDMA and benzodiazepines (BZDs: alprazolam, clonazepam, delorazepam, diazepam, flurazepam, lorazepam, midazolam, nitrazepam, oxazepam, temazepam, and triazolam) were analyzed by gas chromatography–mass spectrometry (a Focus gas chromatograph coupled with an ISQ mass spectrometry, Thermo Electron Corp., Milan, Italy) in selective ion monitoring mode.

To measure alcohol intake in the hair samples, ethyl β-D-6-glucuronide (EtG) was chosen as a specific marker, and its extraction and analysis were carried out according to a fully validated method [19]. EtG chromatographic analysis was carried out using a 7890B Agilent gas chromatograph (Agilent Technologies, Santa Clara, CA, United States) coupled to an Agilent 7000C triple quadrupole mass spectrometer detector with an electron impact ion source. Data were acquired in the multiple reaction monitoring mode. The method was fully validated and routinely applied in our laboratory.

### 2.3. Statistical Analysis

Descriptive statistics were performed using frequencies, percentages and frequency tables for categorical variables, as well as medians and interquartile ranges (IQRs) for quantitative variables.

A generalized linear model (GLM) was adjusted for repeated measures (frequency weights) with time dummies, and a variance Poisson function and link-function logarithmic analysis was performed to evaluate the count data of substances over four monitoring time points. 

One-way repeated-measures ANOVA was also performed to investigate EtG concentration (continuous variable). The Shapiro–Wilk W test was used to assess the normality of the distribution of the data. To allow for the possible violation of compound symmetry or sphericity, we considered the Huynh–Feldt, Box and Greenhouse–Geisser approaches.

After the ANOVA, we estimated the predictive margins of the EtG and performed pairwise comparisons (as contrasts) across the EtG monitoring duration, and all tests and confidence intervals were adjusted for multiple comparisons (Tukey’s method). 

A *p*-value of less than 0.05 was considered to indicate statistical significance.

Statistical analyses were performed using STATA 16.1 (Stata Statistical Software: Release 16, College Station, TX, USA).

## 3. Results

In the present study, 30 subjects with SUDs were monitored for alcohol and drug use quarterly during the first wave of the COVID-19 pandemic in Italy (56.7% male, 43.3% female, median age 30). Home confinement lasted from 22 March to 18 May 2020. Two toxicological control measurements were obtained in December 2019 and March 2020, corresponding to the phase before the lockdown. Furthermore, one additional toxicological control was obtained in June 2020 that includes the lockdown period, and the final measurement, which concerned the post-lockdown quarter, was obtained in September 2020.

Hair was the selected modality of analysis, as it provides long-term information on drug use. It is a strong, stable tissue that is less affected by adulterants or short-term abstinence and thus has an advantage over traditional matrices (e.g., blood or urine) of being able to confirm long-term exposure to drugs over a period of weeks to months, depending on the length of the hair collected [20]. Considering that the average hair growth is 1 cm per month, a hair sample that was 3 cm in length allowed us to examine a retrospective time window of approximately 3 months [20]. Moreover, hair sampling is simple, rapid and minimally invasive, and it supports the identification and quantitation of multiple analyses per sample [20].

**Figure 1:**
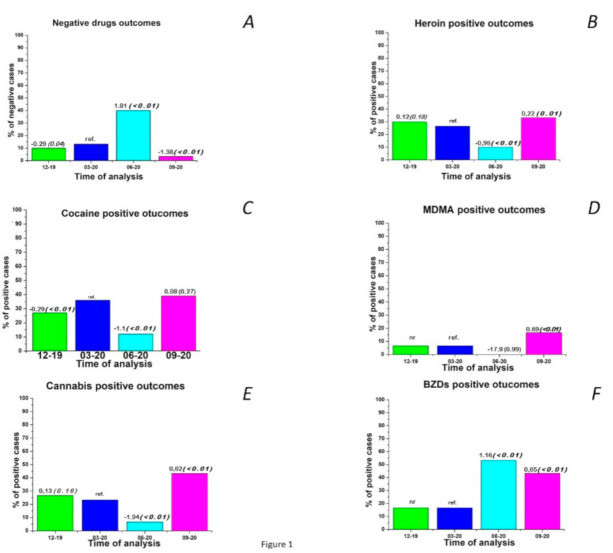
Percentage of samples negative for any drug (**A**), percentage of samples positive for heroin (**B**), cocaine (**C**), MDMA (**D**), cannabis (**E**) and benzodiazepines (BZDs) (**F**) according to the analysis before (December 2019, March 2020 as reference), during (June 2020) and after (September 2020) Italy’s first wave of the COVID-19 pandemic. Coefficients of the generalized linear model (GLM) and relative *p*-values (in brackets) are reported above the histogram bars.

The results obtained by analyzing hair samples show an overall significant reduction in the use of substances during the lockdown period (June 2020) compared with the pre-lockdown control period (March 2020, *p* < 0.01) (Figure 1A).

The percentage of samples positive for heroin decreased from 30% (9 cases) and 26.70% (8 cases) in the two pre-lockdown controls to 10% (3 cases) during lockdown (*p* < 0.01) and then returned to approximately the initial level (33.3%, *p* = 0.01) when the restrictive measures were relaxed (Figure 1B). A similar situation was observed with regard to the percentage of samples positive for cocaine, which was reduced by approximately 30% during the lockdown (*p* < 0.01), and then returned to approximately pre-lockdown levels (43.3%) in September 2020 (*p* = 0.27) (Figure 1C). The percentage of samples positive for MDMA, which was initially very low (6.7%), decreased to 0 during the lockdown and then increased to 16.7% (*p* < 0.01) after the restrictions were lifted (Figure 1D). The percentage of samples positive for cannabis also decreased considerably from 26.7% (8 cases) and 23.3% (7 cases) in December 2019 and March 2020, respectively, to 6.7% (2 cases, *p* < 0.01) during home confinement and then increased in September to slightly higher levels (43.3%, 3 cases, *p* < 0.01) than before the lockdown (Figure 1E). Interestingly, BZDs consumption followed an opposite pattern to that of the most commonly abused drugs monitored here. In fact, the percentage of samples positive for BZDs ranged from 16.7% (5 cases) in the period before the lockdown to 53.3% (16 cases, *p* < 0.01) during the lockdown and remained high (43.3%, 13 cases, *p* < 0.01) even after the lockdown (Figure 1F). It should be emphasized that the BZDs were not drugs prescribed to the patients included in the study, so their intake represents illicit use in all cases.

Specifically (Table 1), the overall trend of drug intake during the considered interval shows that 11/30 (37%) patients switched from single-drug use in the two pre-lockdown period controls to poly-drug use in the post-lockdown period. Six patients were poly-drug addicts in the first two controls and remained so in the subsequent period.

A total of 24/30 (80%) patients in the post-lockdown control resumed the use of the substance for which they initially tested positive before the lockdown period. Interestingly, among these individuals, 15/24 (63%) added the use of new substances, with BZDs present in more than half of the cases. Two patients, who were negative for any drug use in the pre-lockdown periods, were found to be positive for BZDs in the following two periods. In 9/30 (30%) cases, BZDs replaced the use of the previously used drug before the lockdown period. 

A pattern of consumption similar to that found for BZDs was also observed for alcohol, which, in the present study, was monitored by measuring the variations in EtG, a useful biomarker for detecting alcohol abuse. EtG is a nonoxidative, non-volatile, stable, minor direct ethanol metabolite that forms in the liver as the result of ethanol and glucuronic acid conjugation and can be collected in several body fluids and tissues [21]. EtG is also incorporated into hair, which makes its analysis an effective and non-invasive method to retrospectively monitor alcohol consumption over a long period of time (weeks/months) [21]. Based on internationally adopted cut-off concentrations, abstinence from alcohol can be verified (EtG in hair < 7 pg/mg), and chronic excessive drinking with a consumption of 60 g or more of ethanol per day can be detected (>30 pg/mg). EtG concentrations between 7 and 30 pg/mg in hair are regarded as a strong indicator of regular alcohol consumption [22,23].

**Figure 2:**
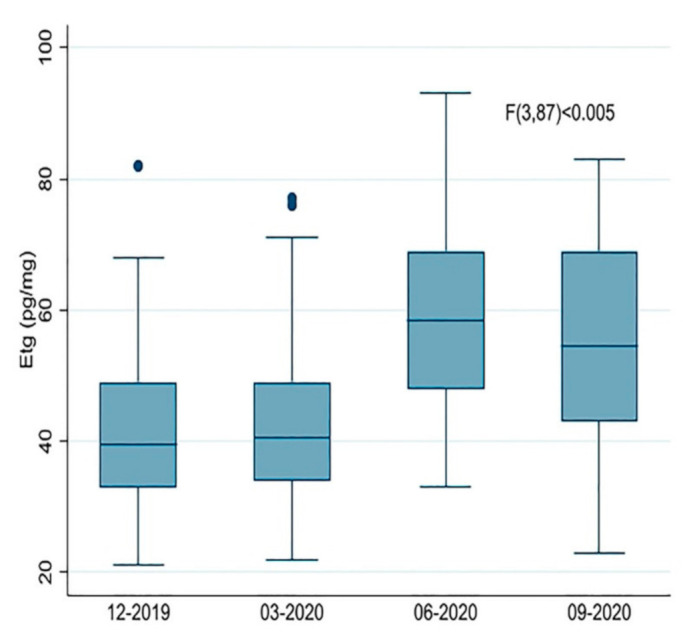
Boxplot of the ethyl β-D-6-glucuronide (EtG) values measured in hair before (December 2019 and March 2020), during (June 2020) and after (September 2020) Italy’s first wave of the COVID-19 pandemic and relative lockdown measures.

Interestingly, the EtG values detected here increased significantly (*p* < 0.005) from the median values of 39.5 pg/mg (IQR 16) and 40.5 pg/mg (IQR 15) in the pre-lockdown periods, to 58.5 pg/mg (IQR 21) in the lockdown period, and remained high with a slight decrease (*p* = 0.05) to 54.5 pg/mg (IQR 26) in the post-lockdown period (Figure 2, Supplementary Table S1 and Supplementary Figure S1). The mean percentage variation between June 2020 and March 2020 was approximately 42%, and higher increases in the EtG values were observed in cannabis and cocaine users (46.2% and 45.5%, respectively).

## 4. Discussion

The experimental data presented here show an overall change in the pattern of drug use as a result of the unusual situation linked to the COVID-19 pandemic.

The strict limitations imposed on the movement and gatherings of people as a rapid response to the pandemic have greatly restricted social opportunities to use drugs [24]. The restrictions affected both the locations where those who use drugs generally gather and their ability to socialize. Pubs and clubs have been closed, and festivals have been cancelled. Additionally, the movements of people and social interactions have been severely limited by the implementation of quarantine and confinement measures [24].

These situations have commonly been cited as leading reasons for the decrease in the use of recreational drugs, such as MDMA and cocaine, as demonstrated in this study, which are typically related to nightlife and party settings [24]. Unlike the findings for cocaine and MDMA, a less marked effect on cannabis use during the lockdown has been reported in some studies [24,25].

The results from the present study show a general tendency to move from the use of a single substance to polysubstance use in the post-lockdown period. Furthermore, 80% of those who abandoned the use of illicit drugs during the lockdown period later returned to using the primary drug, and most of these individuals combined it with other substances. These results suggest that there was full drug availability due to the reopening of the clandestine market right after the lockdown.

By analyzing the trends of individual drugs, our results show a significant decrease in cannabis use during the lockdown, although it tended to return to pre-lockdown levels in the post-lockdown period. A decrease in cannabis use could reflect a reduced availability of the drug in times of social lockdown since the borders were closed, dealers had a harder time going around unnoticed, and prices increased due to increased demand [25]. Our data also show a reduction in heroin use during lockdown in the high-risk drug use population in Italy in this study. In some countries, it was reported that opioid users experienced a sharp decline in their primary sources of income (including panhandling and sex work) and a restriction in their ability to access drugs from their usual drug dealers [24]. This crisis led some heroin users to access drug treatment services, while others switched to using other more readily available substances, such as alcohol and BZDs [24]. In fact, in our study, 30% of the patients replaced the use of the drug that they previously used with BZDs during the lockdown. During this period of home confinement, drug users may not have been looking for substances that are often consumed in social settings, but rather for psychotropic drugs that are more often consumed alone [26].

The trends highlighted in this study show an overall increase in the consumption of BZDs, which has remained high, even in the post-lockdown period. BZDs enhance the effect of the neurotransmitter gamma-aminobutyric acid (GABA) at the GABA_A_ receptor, resulting in sedative, hypnotic (sleep-inducing), anxiolytic, anticonvulsant and muscle-relaxant properties [27].

BZDs have a high potential for abuse and misuse, and they are typically co-abused in patients with SUDs, in whom the most frequent primary abuse drugs are opioids and/or alcohol. BZDs are misused to enhance the euphoric effects of other drugs, to reduce the unwanted effects of drugs, such as insomnia due to stimulant use and to alleviate withdrawal symptoms between doses [27]. The side effects of BZDs are common and include blurred or double vision, headache, vertigo, nausea and vomiting, tremor and depression [27]. Elderly patients are more likely to develop significant adverse effects [27].

In our study, the increased use of BZDs was associated with an increased use of alcohol, both during and after lockdown. Despite extensive research, the precise mechanism of action of ethanol remains not fully understood [28]. However, ethanol has shown a similarity in its effects to positive allosteric modulators of the GABA_A_ receptor, such as BZDs, barbiturates, and various general anesthetics with which alcohol has a synergistic interaction as a CNS depressor [28].

Changes in patterns of alcohol use have been fairly commonly reported in the EMCDDA Trendspotter briefings [24], including drinking more frequently, consuming greater quantities of alcohol and drinking alone. Increased alcohol consumption is commonly observed after a crisis [29]. There are several reasons why alcohol consumption may increase during the COVID-19 pandemic. These include boredom and disruption to routines caused by the lockdown, the threat of the disease, changes to life circumstances and associated distress [30].

A study of 754 adults from the USA demonstrated that psychological distress caused by the COVID-19 pandemic was associated with increased alcohol consumption, whereas the perceived threat from the virus itself was not associated with increased alcohol consumption [31]. Other reasons people drink during a crisis include the inhibiting effect of alcohol on the nervous system offering temporary relief from emotions, anxiety, anger, sleep disorders, depression and post-traumatic stress disorders associated with a lockdown [30].

Interestingly, the consumption of all the substances analyzed (heroin, cocaine, MDMA and cannabis) at the end of the lockdown returned to levels similar to those found prior to the lockdown, except for alcohol and BZDs, the consumption of which has remained at the higher levels reached during lockdown. Although patients in the study had fairly high baseline EtG values, since alcohol is among the most frequently reported secondary substance problems for drug addicts, the highly significant increase in alcohol consumption, especially among cannabis and cocaine users, observed during and after the lockdown can be attributed to the effects of the pandemic, as highlighted by other authors [32,33,34].

Periods of isolation and loneliness can have long-lasting negative consequences on individuals’ physical and mental well-being, which can lead to an increase in the consumption of self-care substances such as alcohol and BZDs, both of which are central nervous system depressants that can result in a range of adverse health effects [26,30,31]. Overall, the COVID-19 pandemic could lead to a spike in alcohol abuse, relapses and potentially the development of alcohol use disorders combined with the concomitant abuse of other drugs in at-risk individuals.

Severe short- and long-term physical, psychological and social consequences related to alcohol and/or drug abuse during the COVID-19 pandemic will inevitably put further pressure on drug and alcohol dependence services and the health service in general during the post-pandemic period [15,27,32].

## 5. Conclusions

The health and social consequences of the COVID-19 pandemic are not yet clearly known. Even though it is mostly accepted that lockdown policies are aimed at limiting the virus’s spread, there is increasing concern regarding their collateral impacts, such as on drug and/or alcohol use. The present study monitored 30 patients with SUDs before, during and immediately after the lockdown in Italy following the first wave of COVID-19 and showed clear changes in substance use patterns. In particular, there was an increased tendency to replace illicit drugs with other potentially dangerous, but more easily available, substances such as alcohol and BZDs. This increase in the use of alcohol and BZDs has continued after the lockdown, while the use of illicit drugs has returned to pre-lockdown levels, leading to an elevated risk of developing comorbid psychiatric disorders and other health conditions [35]. Therefore, it is important that governments provide the public with warnings about excessive alcohol consumption during isolation to protect vulnerable individuals [36]. Thus, promoting a much wider adoption of appropriate evidence-based prevention, treatment and recovery strategies needs to be a top public health priority that meets the specific and changing needs of people who use substances during the COVID-19 pandemic. Engagement with people who use drugs should be prioritized in the planning, design and delivery of emergency planning and mitigation strategies.

This study was limited to a population at high risk for alcohol and drug use and monitored a small number of subjects. Therefore, continued monitoring of these patients during the second wave of the COVID-19 pandemic in Italy is proposed to obtain data that can be used to determine whether the observed changes in drug use patterns are stable. It would also be useful to combine experimental data on the consumption of drugs and alcohol in the general population with other reports from toxicology laboratories based on data collected during the COVID-19 pandemic.

## Figures and Tables

**Table 1:** Drugs found in hair before (December 2019 and March 2020), during (June 2020) and after (September 2020) the lockdown measures (findings of specific drugs were highlighted with different colors).

Patient	December 2019	March 2020	June 2020	September 2020
	Drugs Found	Drugs Found	Drugs Found	Drugs Found
1	Cocaine	Cocaine	BZDs	Cocaine
				Cannabis
				BZDs
2	Cannabis	Cannabis	Negative	Cannabis
				Cocaine
3	Heroin	Heroin	BZDs	Heroin
	BZDs	BZDs		Cannabis
				BZDs
4	Negative	Negative	BZDs	BZDs
5	MDMA	MDMA	Negative	MDMA
				Cocaine
6	Cannabis	Cannabis	BZDs	Cannabis
				Cocaine
7	Heroin	Heroin	Heroin	Heroin
	BZDs	BZDs	Cannabis	Cannabis
			BZDs	BZDs
8	Cocaine	Cocaine	Cocaine	Cocaine
				MDMA
9	Cannabis	Cannabis	Negative	Cannabis
10	Heroin	Heroin	Heroin	Heroin
	Cocaine	Cocaine	BZDs	Cocaine
				BZDs
11	Heroin	Heroin	Negative	BZDs
12	Heroin	Heroin	BZDs	Heroin
13	BZDs	Cocaine	Negative	Cocaine
14	Cocaine	Cocaine	Cocaine	Cocaine
	MDMA	MDMA		MDMA
				BZDs
15	Cocaine	Cocaine	Negative	Cocaine
			BZDs	BZDs
16	Heroin	Heroin	Negative	Heroin
	Cannabis	BZDs		BZDs
17	Negative	Negative	BZDs	BZDs
18	Cocaine	Cocaine	Negative	Cocaine
19	Negative	Negative	Negative	Negative
20	Cocaine	Cocaine	Cocaine	Cocaine
			BZDs	
21	Cannabis	Cannabis	Negative	Cannabis
22	Cannabis	Cannabis	Cannabis	Cannabis
			BZDs	
23	Heroin	Heroin	BZDs	Heroin
24	BZDs	Cocaine	BZDs	Cocaine
				Cannabis
25	Cocaine	Cocaine	Negative	Heroin
				MDMA
26	Heroin	Heroin	BZDs	Heroin
	BZDs	Cannabis		Cannabis
				BZDs
27	Heroin	Cocaine	Heroin	Heroin
		BZDs	BZDs	Cannabis
				BZDs
28	Cannabis	Negative	Negative	Cannabis
29	Cannabis	Cannabis	BZDs	Heroin
				BZDs
30	Cocaine	Cocaine	Cocaine	Cocaine
		BZDs		MDMA
				Cannabis

## Data Availability

The data presented in this study are available on request from the corresponding author. The data are not publicly available due to privacy restrictions.

## Supplementary Materials

The following are available online at https://www.mdpi.com/1660-4601/18/4/1967/s1, Figure S1: Predictive margins of EtG with 95% Cls, Table S1: Pairwise comparisons of marginal linear predictions on times of monitoring EtG.
