# Supplementary material for: Changes in Drug Use Patterns during the COVID-19 Pandemic in Italy: Monitoring a Vulnerable Group by Hair Analysis
Source: Int J Environ Res Public Health. 2021 Feb 18;18(4):1967. doi: 10.3390/ijerph18041967 (PMC7922660; doi:10.3390/ijerph18041967)
Supplement: Supplementary file 1 [file ijerph-18-01967-s001.zip › table 1 supplementary.docx]

| **Times of monitoring** | **Contrast** | **P>t** |
| --- | --- | --- |
| 03-2020 vs. 12-2019 | 1.17 | 0.845 |
| 06-2020 vs. 12-2019 | 17.53 | 0.000 |
| 09-2020 vs. 12-2019 | 13.80 | 0.000 |
| 06-2020 vs. 03-2020 | 16.37 | 0.000 |
| 09-2020 vs. 03-2020 | 12.63 | 0.000 |
| 09-2020 vs. 06-2020 | -3.73 | 0.049 |

Table.1 Pairwise comparisons of marginal linear predictions on times of monitoring Etg
