# Supplementary figures and images for: Changes in Drug Use Patterns during the COVID-19 Pandemic in Italy: Monitoring a Vulnerable Group by Hair Analysis
Source: Int J Environ Res Public Health. 2021 Feb 18;18(4):1967. doi: 10.3390/ijerph18041967 (PMC7922660; doi:10.3390/ijerph18041967)

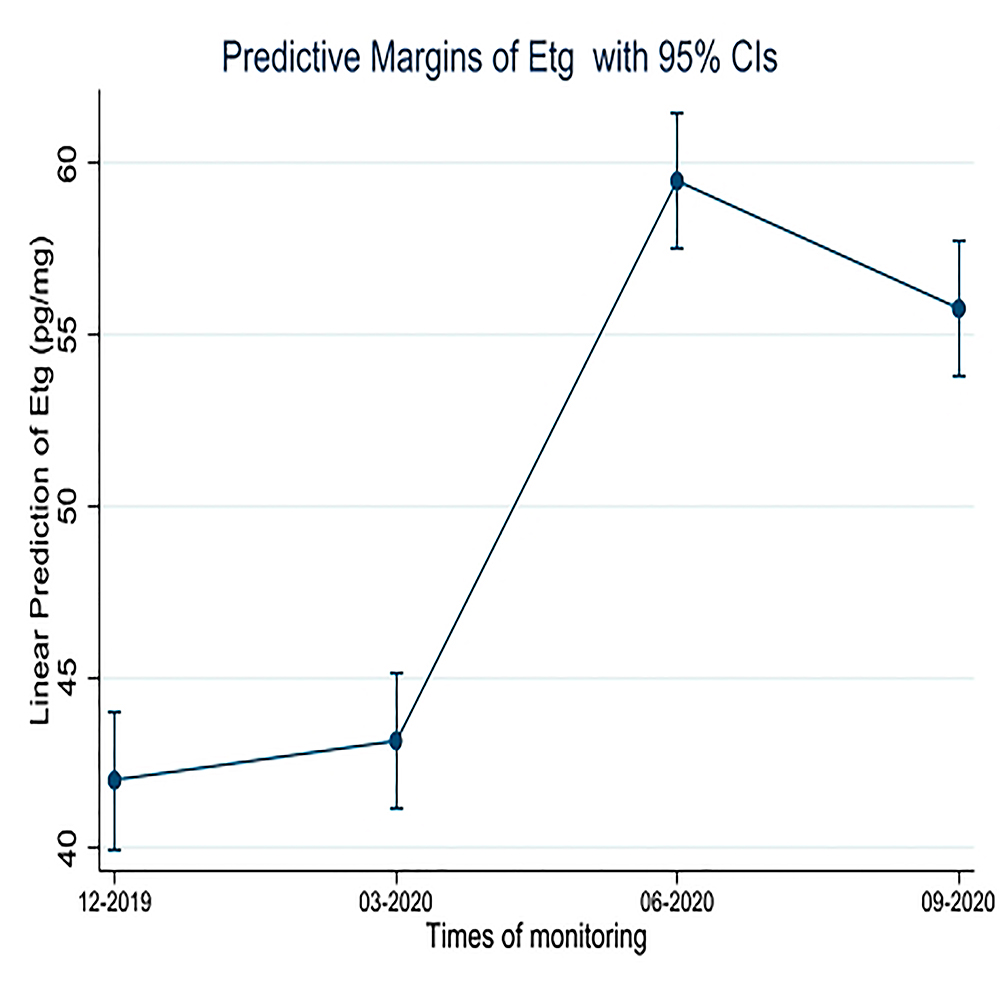

Supplement: Supplementary file 1 [file ijerph-18-01967-s001.zip › Figure 1 supplementary.jpg]
